# Supplementary material for: Iterative improvement in the automatic modular design of robot swarms
Source: PeerJ Comput Sci. 2020 Dec 7;6:e322. doi: 10.7717/peerj-cs.322 (PMC7924708; doi:10.7717/peerj-cs.322)
Supplement: Supplemental Information 3 [file peerj-cs-06-322-s003.zip › argos3/doc/api/standalone/a00580_source.html]

ARGoS: plugins/simulator/entities/omnidirectional\_camera\_equipped\_entity.cpp Source File


- Main Page
- Related Pages
- Namespaces
- Classes
- Files

- File List
- File Members

# plugins/simulator/entities/omnidirectional\_camera\_equipped\_entity.cpp

Go to the documentation of this file.

```
00001 #include "omnidirectional_camera_equipped_entity.h"
00002 #include <argos3/core/simulator/space/space.h>
00003 
00004 namespace argos {
00005 
00006    /****************************************/
00007    /****************************************/
00008 
00009    COmnidirectionalCameraEquippedEntity::COmnidirectionalCameraEquippedEntity(CComposableEntity* pc_parent) :
00010       CEntity(pc_parent) {
00011       Disable();
00012    }
00013 
00014    /****************************************/
00015    /****************************************/
00016 
00017    COmnidirectionalCameraEquippedEntity::COmnidirectionalCameraEquippedEntity(CComposableEntity* pc_parent,
00018                                                                               const std::string& str_id,
00019                                                                               const CRadians& c_aperture,
00020                                                                               const CVector3& c_offset) :
00021       CEntity(pc_parent, str_id),
00022       m_cAperture(c_aperture),
00023       m_cOffset(c_offset) {      
00024       Disable();
00025    }
00026 
00027    /****************************************/
00028    /****************************************/
00029 
00030    void COmnidirectionalCameraEquippedEntity::Init(TConfigurationNode& t_tree) {
00031       try {
00032          CEntity::Init(t_tree);
00033          GetNodeAttribute(t_tree, "aperture", m_cAperture);
00034          GetNodeAttribute(t_tree, "offset", m_cOffset);
00035       }
00036       catch(CARGoSException& ex) {
00037          THROW_ARGOSEXCEPTION_NESTED("Error initializing the omnidirectional camera equipped entity \"" << GetId() << "\"", ex);
00038       }
00039    }
00040 
00041    /****************************************/
00042    /****************************************/
00043 
00044    REGISTER_STANDARD_SPACE_OPERATIONS_ON_ENTITY(COmnidirectionalCameraEquippedEntity);
00045 
00046    /****************************************/
00047    /****************************************/
00048 
00049 }
```

---

Generated on 10 Jul 2018 for ARGoS by 
 1.6.1
